# Specialist vs. General Practitioner

**Published:** 1896-05

**Authors:** 


### Selections and Abstracts.

### SPECIALIST VS. GENERAL PRACTITIONER.

General practitioners, as a rule, regard specialists in very much the same light as the public regards the medical profession as a whole, namely, as an inevitable evil to whose support they must contribute, but at whom they have the compensatory privilege of leveling their shafts of satire and invective. This sentiment may be partly due to the source from which the specialist has evolved. Going back to the middle ages, we find the first specialist, the surgeon acting as the mechanically skillful but unrespected assistant of the physician. The Chamberlens, to whom we owe the invention of the obstretric forceps, kept their discovery a secret for three generations, and advertised in the most quackish terms their peculiar superiority in relieving the parturient woman. The radical cure of hernia, the operations on hemorrhoids, the special care of the eyes and ears, originated among peripatetic charlatans. The genito-urinary and the nervous specialist have differentiated the functions of the healer of "clap" and the restorer of "lost manhood." Dermatology has but recently been claimed from the hands of the charlatan, and the chiropody and orificial surgery are just beginning to be recognized as fields for legitimate medical specialists, while one of the greatest of foreign authorities on digestive diseases is still under a cloud on account of unprofessional conduct in earlier life. It is humiliating to be obliged to confess that the medical specialist has but followed in the footsteps of the pioneer quack, yet too much credit can scarcely be given to the former for having so soon effaced the tracks of the latter.

Unfortunately, however, the general practitioner still regards the specialist as a parasite, grudges him his source of livelihood, and reluctantly yields to him only the most refractory cases, or implores his charity for the poor while the rich are subjected to experiments until his patience is exhausted. We appreciate the fact that there are specialists who are dishonest, who have essayed the niceties of an art whose rudiments they do not comprehend, who deserve the epithets "fakes," "bluffers," and other more refined but less expressive terms which have been bestowed upon them. We believe, however, that such instances are the exception, and shall proceed with the discussion of this topic without any qualifications which the allowance of dishonesty would require.

The general practitioner too often sums up his relation to the specialist as follows: "I have sent patients to that man; he is dependent on the favors of men like myself, and what does he do in return?" Superficially considered, it does seem as if the obligation were all on the side of the specialist, but a little analysis will show that it is mutual. So far as requirements of preliminary and medical training are concerned, including the expenditure of time and money, specialist and general practitioner are on the same footing before the law. The support of medical societies, of medical literature, of social and benevolent enterprises in the profession, is drawn equally from all. The specialist has, in nearly every case, been to a much greater expense for post-graduate study; he is compelled to exert greater effort to keep abreast of new discoveries; his books, instruments and office service are more expensive, yet he has voluntarily renounced his right to practice medicine except in a limited field. He may not reciprocate favors by sending patients to all those who have referred cases to him, but his more or less public announcement that he wishes only cases of such a nature leaves a greater demand for the services of those who say, "What has he done for me?" We must not forget that many consult a specialist who would not go to the general practitioner, and whom the latter could not relieve if they did seek him. The general practitioner who expects the specialist to reciprocate in the referring of patients, may as well pause to ask whence the specialist is to draw such patients. Unless in the transitional stage from general practice to specialism, the specialist cannot refer patients to the general practitioner unless we count the very occasional and not very desirable cases who ring the door bell of the first doctor whose sign attracts their eyes, or unless patients sent from one physician are diverted into the practice of another, or unless the specialist is taking patients who do not belong to him for the sake of cancelling debts of courtesy. It must be remembered, therefore, that the best evidence that a specialist is acting in good faith and that he is reciprocating the favors of the profession as a whole, is the fact that he is not in a position to return in kind the referring of a patient.

Undoubtedly the lot of a specialist is more desirable than that of the general practitioner. This is apt to be the case with any new position which only comparatively few can reach after patient preparation, prolonged effort, and the demonstration of unusual brilliancy—or unusual capacity for hard work, which is sometimes mistaken for brilliancy. The specialist is less exposed to the inclemency of the weather, his fees are larger and more certain, but, on the other hand, his actual hours of work are at least as many and less diversified; he may save the time of transit which the general practitioner must waste, but he lacks the recreation which the latter can intersperse with professional cares. If his fees are larger, and more promptly paid, he must show more attention to his patients and must spend more time in general study and experimentation, and in the long run, he must demonstrate his ability to obtain better results. And finally, specialists, though comprising but a small part of the total medical profession, are called upon for the greater part of the gratuitous attendance upon physicians and their families.

Whenever we find a specialist overstepping the proper bounds of his practice, the reason is one of two factors which act and re-act on each other. Either the specialist is unfair to the rest of the profession in following the plan of keeping all he gets and getting all he can, or the general practitioners are not sending to the specialist enough patients to keep him busy in his own specialty. We are inclined to think that the blame rests more often on the general practitioners, since, every time the specialist accepts a patient who does not belong to him, he admits that he is not thoroughly successful in his own line, and fails to advertise his specialty in a legitimate manner to a possible clientele. The general practitioner, on the other hand, may secure a confinement or an accident case through demonstrating his skill with pneumonia, and he is not under obligations to turn away any case which does not present peculiar difficulties calling for special apparatus or special experience for its diagnosis and treatment.

We believe that, as for all attempts at codifying medical ethics, the Golden Rule affords the best solution of the problems which arise in settling conflicts between specialist and general practitioner. The general practitioner who calls every gastric disease dyspepsia and treats all impartially with bismuth and pepsin, the orificialist who essays to relieve catarrhal headache by stretching the spincter ani, the gynæcologist who removes the ovaries of a hysterical girl, the nervous specialist who uses hypnotic suggestion to cure a case of bronchitis which he calls spasmodic asthma, the men who are so consistent in their limitations of practice that the oculist sends his patient to the physician for a cathartic pill, and the gynæcologist and the surgeon bow to each other across the brim of the pelvis while the patient is mulcted of a double fee—to all such men and to others who choose to quibble over the mutual rights of specialist and general practitioner, we can [illegible] ly say, cultivate a conscience—*Medical and Surgical Reporter*.

